# Oral maxillofacial surgeons and Orthodontists’ perceptions about anterior inferior crowding and indications of mandibular third molar extraction

**DOI:** 10.4317/medoral.26218

**Published:** 2023-10-12

**Authors:** Christian Recchioni, Emyr Stringhini Junior, Juliana Cama Ramacciato, Luciana Butini Oliveira

**Affiliations:** 1 School of Dentistry, Faculdade São Leopoldo Mandic, Campinas, SP, Brazil

## Abstract

**Background:**

There are still many doubts about anterior inferior crowding and indications of mandibular third molar extraction, although it is very studied subject in the literature. The aim of this study was to evaluate the perceptions of oral maxillofacial surgeons (OMFSs) and orthodontists about anterior inferior crowding and indications of mandibular third molar extraction.

**Material and Methods:**

A web-based survey was developed and sent to professionals in order to collect their opinion about the fact that third lower molars cause crowding and questions about the indication of third molars for orthodontic treatment. Descriptive analysis was performed and Chi-square or G tests were applied with a 95% confidence interval.

**Results:**

The study included a total of 218 participants, of whom 115 were OMFSs and 103 were orthodontists. The results showed that 56.5% of OMFSs and 35.0% of orthodontists believe that the lower third molars cause anterior inferior crowding (*p*<0.001). A total of 91.3% of OMFSs and 70.9% of orthodontists indicate the extraction of lower third molars to aid orthodontic treatment (*p*<0.001).

**Conclusions:**

It can be concluded that in being an oral maxillofacial surgeon, a higher odds ratio is observed to consider that lower third molars cause dental crowding compared to those who are orthodontist. The indication of exodontia of lower third molars for orthodontic treatment was more frequent among OMFSs when compared to orthodontists.

** Key words:**Third molar, malocclusions, dentists’ knowledge, oral surgery.

## Introduction

Various etiologies are associated with crowding, such as: dental factors, involving the size of the crown, loss of dental arch length, periodontal status and early loss of primary teeth; skeletal factors, involving growth of the maxilla and malocclusions; and general factors, involving age and gender (1). Many theories have attempted to explain the reasons of the lower incisor crowding. It remains unknown the degree of interaction of different factors and how to analyze them *in vivo* (2). Some authors reported that mandibular anterior crowding is not clarified in the literature and they suggested that it represents the break of tensegrity in the dental arch (3).

In orthodontic therapy, third molars generate controversies, considering their capacity for causing crowding and relapses in the post-orthodontic therapy period. It has been hypothesized that at the stage of eruption, they may exert anterior force (4,5). Some authors were emphatic in affirming that there was no scientific evidence that proved the relationship between mandibular third molars and anterior inferior crowding (1,2,6-8). On the other hand, a systematic review identified studies and reviews related to the effect of the lower third molars on the lower dental arch crowding and the authors concluded that the results are quite contradictory. Some studies support the opinion that lower third molars cause teeth crowding, the others confirm conversy (9). Furthermore, a recent systematic review concluded that there is insufficient evidence available for determining whether asymptomatic disease-free impacted wisdom teeth should be removed or retained (10).

There is a scarcity of previous studies that have compared the opinion of surgeons and orthodontists about the indication of third molar extraction. Lindauer *et al*. (11) verified that there was a significant disagreement among both orthodontists and oral and maxillofacial surgeons, regarding the fundamental issues underlying the role of third molars in dental crowding. A smaller percentage of orthodontists than surgeons believed that maxillary and mandibular third molars produce anterior forces during eruption. Similarly, orthodontists were less likely to think that maxillary and mandibular third molars cause anterior crowding and were therefore less likely to recommend prophylactic removal of maxillary and mandibular third molars to prevent crowding. Surgeons were more likely to "generally" or "sometimes" (56.9%) recommend prophylactic removal of mandibular third molars to prevent crowding, whereas orthodontists more often said that they "rarely" or "never" (64.4%) recommend it.

Conversely, another previous study concluded that Italian oral surgeons and orthodontists have the same opinion on the role of the third molar in causing anterior crowding. The majority of both groups of clinicians did not consider their preventive extraction useful in order to prevent anterior crowding (12). Therefore, the aim of this study was to evaluate the perceptions of maxillofacial surgeons and orthodontists about anterior crowding and indications of mandibular third molar extraction.

## Material and Methods

- Ethical consideration and report

The study was approved by the Ethics Committee of São Leopoldo Mandic Institute and Research Center under registration number 4600713, Campinas, São Paulo, Brazil. All participants gave written informed consent prior to the use of their data. This manuscript was structured to best-fit to guidelines for reporting observational studies (13).

- Study design, sample and data collection

A cross-sectional web-based survey was conducted with a sample of orthodontis and OMFSs in a period of 6 months in 2022. Participants were recruited by convenience sampling through data obtained from Regional Council of Dentistry and the sample was categorized according to professional dental specialty. Instructions on the research objectives and how to complete the questionnaire was given. Participants self-filled the study questionnaire on Google Forms. The survey questions were design to 1) evaluate the professionals’ opinion about the fact that third lower molars cause crowding; 2) assess the indication and the moment of extraction of third molars for orthodontic treatment, and for those who refer the patient to extraction.

- Statistical analysis

The results obtained from questionnaires were tabulated and descriptive analysis was performed. In addition, Chi-square or G tests were applied in order to investigate associations of dependent variables opinion about the fact that lower third molars cause crowding and the indication and moment of extraction of third molars for orthodontic treatment with the independent variable specialty of the professional. The SPSS 23 (SPSS INC., Chicago, IL, USA) and BioEstat 5.0 (Fundação Mamirauá, Belém, PA, Brazil) softwares were used for all analyses. The significance level was set at 0.05.

## Results

A total of 218 specialists completed the questionnaire. There were 115 (52.8%) responses from OMFSs and 103 responses from orthodontists. Among the participants in the research, 101 (53.7%) answered that they considered that lower third molars cause dental crowding. In the sample of 103 orthodontists, 36 (35.0%) reported that third molars were the cause of anterior crowding, while in the universe of 115 OMFSs, 65 (56.5%) professionals that third molars were the cause of anterior crowding (Table 1). There was a statistically significant association between the 2 specialty groups (*p*=0.001, Chi-square test). Specifically, in the group of OMFSs, an odds ratio 2.42 times higher was observed, with a confidence interval (95%) of 1.40 to 4.18, of considering that lower third molars cause anterior crowding compared to orthodontists.

Surprising, when asked if they indicate extraction of lower third molars for orthodontic treatment, 178 (81.7%) answered positively. For the sample of 103 orthodontists, 73 (70.9%) reported indicating the referred extraction for orthodontic treatment, while among the 115 OMFSs, there were 105 (91.3%) positive responses (Table 1). There was a statistically significant association between the 2 groups and the indication of lower third molar extraction for orthodontic treatment (*p*<0.001).

**Table 1:**
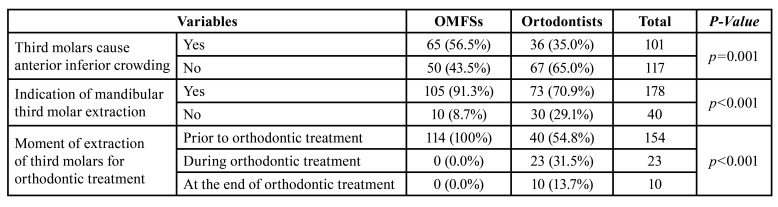
Frequency of responses indicated by orthodontists and OMFSs about about anterior inferior crowding and indications of mandibular third molar extraction.

As an oral maxillofacial surgeon, there is a 4.31 times higher odds ratio, with a confidence interval (95%) of 1.99 to 9.27, of indicating extraction of lower third molars for orthodontic treatment when compared to those who are orthodontists.

Most of the 218 respondents, 154 of them (70.6%), reported indicating the extraction of lower third molars prior to orthodontic treatment, while 10.6% and 4.6% reported being at the end of or at the end of orthodontic treatment, respectively. The specialty was significantly associated with the choice of the moment of extraction of lower third molars for orthodontic treatment (*p*<0.001). The indications of extraction of lower third molars during or even at the end of orthodontic treatment were made exclusively by orthodontists.

All OMFSs indicated that they themselves do the extraction of lower third molars, while only 23.3% of the Orthodontists reported that they themselves perform the extraction. There were also orthodontists who said they referred to professors of oral surgery in undergraduate courses (10.9%) and others who signaled to indicate the patient to an institution that has a course of oral and maxillofacial surgery (1.4%).

## Discussion

In this study the perceptions of orthodontists and OMFSs about anterior crowding and indication of mandibular third molar extraction was evaluated. The main results demonstrated that 35.0% of orthodontists and 56.5% of surgeons believe that the lower third molars cause anterior crowding. A total of 70.9% of orthodontists and 91.3% of surgeons indicate the extraction of lower third molars to aid orthodontic treatment. For the sample of 103 orthodontists, 73 (70.9%) reported indicating the referred extraction for orthodontic treatment, while among the 115 OMFSs, there were 105 (91.3%) positive responses.

Despite the different opinions expressed by both groups of professionals surveyed in this study on the role of the third molar in causing anterior inferior crowding, the majority of orthodontists and OMFSs indicate the extraction of lower third molars. Conversely, a previous study found differences in orthodontists' and oral and maxillofacial surgeons' beliefs about the association between third molar eruption and the development of crowding (12). Some authors studied the influence of graduation year and verified that more recently graduated orthodontists were less likely to recommend prophylactic removal of third molars to prevent crowding, and surgeons were more likely to recommend removal if they graduated in the 1970s or 1980s (13).

The indication for extractions is much more frequent among OMFSs than among orthodontists. It is important to mention that when indicanting extraction of third molars, dentists should have a justifiable reason. In addition, a cost and benefit analysis should be carried out in order to justify this conduct. However, a previous study concluded that general dentists frequently recommended removal of third molars for reasons not related to pathology or symptoms, but rather to prevent future problems (14). On the other hand, orthodontists have opted for the preservation of the mandibular third molars, associated with the use of orthodontic splinting for the purpose of maintaining the stability of the mandibular anterior teeth (1,3,12,15).

A previous study evaluated the ability of OMFSs and orthodontists to predict third molar eruption by examining a simple panoramic radiograph in cases where full spontaneous eruption occurred. The authors concluded that despite a remarkable agreement for third molar prognosis, orthodontists and OMFSs were unable to predict mandibular third molar eruption by examining a simple panoramic radiograph. Both group of professionals indicated extractions of a considerable number of spontaneously erupted asymptomatic teeth (16). The ability of orthodontists and OMFSs in predicting spontaneous eruption of mandibular third molar using panoramic serial radiographs was also evaluated by Libdy *et al*. (17) and the results also confirmed that the orthodontists and OMFSs were not able to predict the eruption of mandibular third molar that have erupted spontaneously. Another similar study also evaluated the predictive capacity of orthodontists and OMFSs in antecipating the process of impaction or eruption of lower third molars through exanation of serial panoramic radiographs. Both groups of professionals seem unable to predict spontaneous eruption or impaction of the third molars (18).

The risk of both trans- and post-operative accidents and complications (10% on an average) must be analyzed and considered. The risks and benefits must be analyzed in surgical planning, particularly when they are associated with pathologies that justify surgical intervention. These may be influenced by age, gender, position, and degree of impaction, as well as noble anatomic structures (19). Anatomical, radiological and operative variables appear to be important factors in the assessment of surgical difficulty in the extraction of lower third molars (20).

Some researches evaluated the decision making among Israeli, Eastern European, and South American dental school graduates in oral surgery issues during a military dental convention. They recommended that further postgraduate education in this area is warranted due to the fact that the decision making regarding third molar treatment is not evidence-based and is not rational (21). Our results also encourage the practioners to re-evaluate their view about anterior crowding and indications of mandibular third molar extraction considering the available evidences. Most of the included studies in a previous systematic review do not support a cause-and-effect relationship between the third molars and the development of anterior tooth crowding ; therefore, third molar extraction to prevent anterior tooth crowding or postorthodontic relapse is not justified (22). In addition, another study investigated if there is evidence justifying the prophylactic extraction of third molars and their study demonstrated the lack of studies on which to base adequate clinical decisions regarding indications for the prophylactic extraction of third molars (23).

Moreover, a recent systematic review also concluded that there is insufficient evidence available for determining whether asymptomatic disease-free impacted wisdom teeth should be removed or retained. Although retention of asymptomatic disease-free impacted wisdom teeth may be associated with increased risk of periodontitis affecting adjacent second molars in the long term, the evidence has a very low level of certainty. According to the authors, given the current lack of available evidence, patient values should be considered and clinical expertise used to guide shared decision-making with people who have asymptomatic disease-free impacted wisdom teeth. If the decision is made to retain these teeth, clinical assessment at regular intervals is advisable, in order to prevent undesirable outcomes (10).

This study presents as limitation the type of convenience sampling. Future studies with a large sample are needed to explore the influence of year of graduation, age of the professionals and the assess to the scientific evidences that facilitates an implementation of up to date knowledge in the clinical practice.

## Conclusions

It can be concluded that in being an oral maxillofacial surgeon, a higher odds ratio is observed to consider that lower third molars cause dental crowding compared to those who are orthodontist. The indication of exodontia of lower third molars for orthodontic treatment was more frequent among OMFSs when compared to orthodontists. Our results also encourage the practioners to re-evaluate their view about anterior crowding and indications of mandibular third molar extraction considering the available evidences in the literature.
